# Synthesis, antitumor activity, and molecular docking study of 2-cyclopentyloxyanisole derivatives: mechanistic study of enzyme inhibition

**DOI:** 10.1080/14756366.2020.1740695

**Published:** 2020-03-18

**Authors:** Walaa M. El-Husseiny, Magda A.-A. El-Sayed, Adel S. El-Azab, Nawaf A. AlSaif, Mohammed M. Alanazi, Alaa A.-M. Abdel-Aziz

**Affiliations:** a Department of Pharmaceutical Organic Chemistry, Faculty of Pharmacy, Mansoura University, Mansoura, Egypt; b Department of Pharmaceutical Chemistry, Faculty of Pharmacy, Horus University, New Damietta, Egypt; c Department of Pharmaceutical Chemistry, College of Pharmacy, King Saud University, Riyadh, Saudi Arabia

**Keywords:** Synthesis, 2-cyclopentyloxyanisole scaffold, antitumor activity, enzyme inhibition assay; docking study

## Abstract

A series of 24 compounds was synthesised based on a 2-cyclopentyloxyanisole scaffold **3–14** and their *in vitro* antitumor activity was evaluated. Compounds **4a**, **4b**, **6b**, **7b**, **13**, and **14** had the most potent antitumor activity (IC_50_ range: 5.13–17.95 μM), compared to those of the reference drugs celecoxib, afatinib, and doxorubicin. The most active derivatives **4a**, **4b**, **7b**, and **13** were evaluated for their inhibitory activity against COX-2, PDE4B, and TNF-α. Compounds **4a** and **13** potently inhibited TNF-α (IC_50_ values: 2.01 and 6.72 μM, respectively) compared with celecoxib (IC_50_=6.44 μM). Compounds **4b** and **13** potently inhibited COX-2 (IC_50_ values: 1.08 and 1.88 μM, respectively) comparable to that of celecoxib (IC_50_=0.68 μM). Compounds **4a**, **7b**, and **13** inhibited PDE4B (IC_50_ values: 5.62, 5.65, and 3.98 μM, respectively) compared with the reference drug roflumilast (IC_50_=1.55 μM). The molecular docking of compounds **4b** and **13** with the COX-2 and PDE4B binding pockets was studied.

**Highlights**
- Antitumor activity of new synthesized cyclopentyloxyanisole scaffold was evaluated.
- The powerful antitumor 4a, 4b, 6b, 7b & 13 were assessed as COX-2, PDE4B & TNF-α inhibitors.
- Compounds 4a, 7b, and 13 exhibited COX-2, PDE4B, and TNF-α inhibition.
- Compounds 4b and 13 showed strong interactions at the COX-2 and PDE4B binding pockets.

## Graphical Abstract

**Figure:**
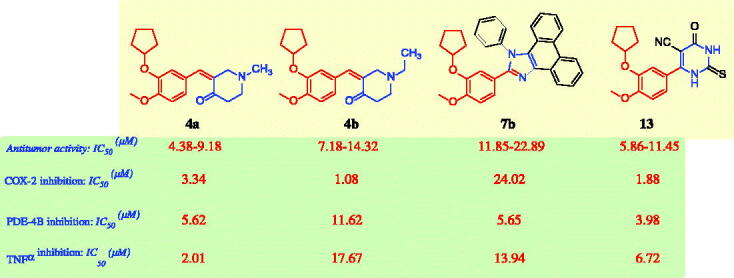

## Introduction

Cancer, the uncontrolled growth of cells that invade adjacent healthy tissues, is the most fatal disease in the world^1^. Therefore, the design and synthesis of new molecules with promising and potential antitumor activity is of great importance^1–10^. The clinical use of drug combinations has led to various side effects, whereas the use of single molecules that target multiple molecular mechanisms is the currently preferred therapeutic strategy and is under investigation by medicinal chemists^11–13^.

**Figure 1.**
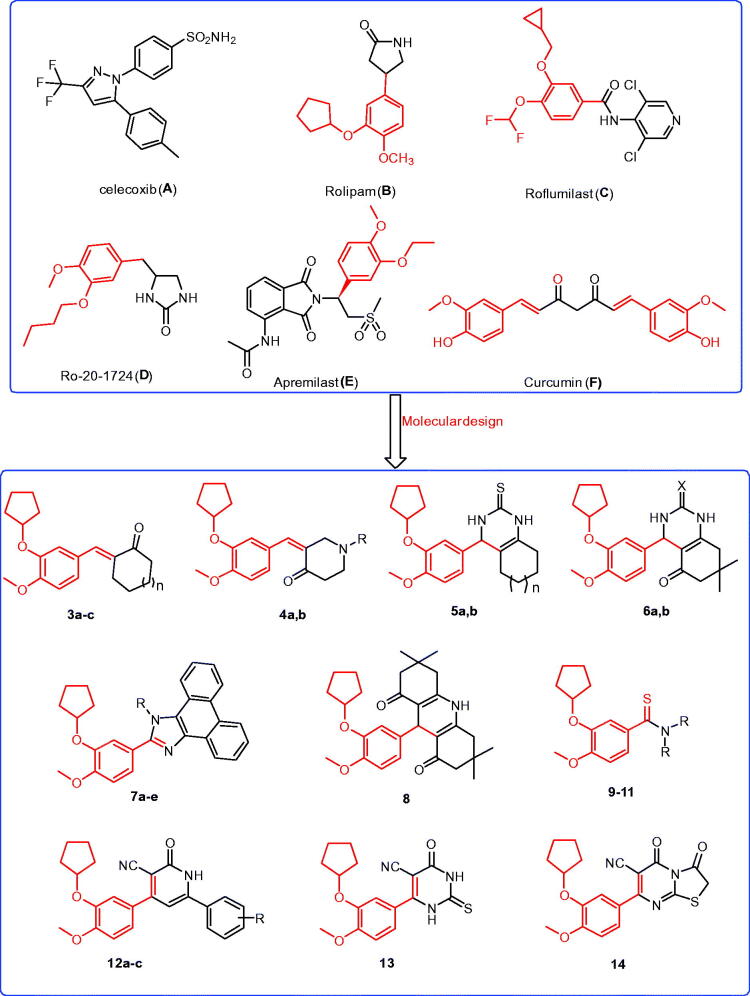
The structures of the reported antitumor agents (**A–F**) with COX-2 or PDE4 and the designed compounds **3–14**.

Cyclooxygenase-2 isoenzyme (COX-2) inhibitors, such as celecoxib (**A**; Figure 1), have been reported to have antitumor activities^8^^,^^14^^,^^15^. The COX-2 isoenzyme is overexpressed in numerous human cancers, such as breast, lung, hepatocellular, gastric, ovarian, prostate, and colon cancers^8^^,^^14–16^. There are two anticancer mechanisms associated with COX-2 inhibition: the first, termed the COX-2-dependent anticancer mechanism, is selective inhibition with the restoration of normal apoptosis; the second is the COX-2-independent mechanism, which occurs through the induction of apoptosis or inhibition of cell proliferation^17^. These results indicated that COX-2 enzyme inhibition was an interesting molecular target for the treatment of cancer^8^^,^^14–17^. In addition, phosphodiesterase isoenzyme 4 (PDE4) is responsible for inactivation and hydrolysis of 3′,5′-cyclic adenosine monophosphate (cAMP) and subdivided into four subtypes, PDE4A to PDE4D^18–20^. The secondary messenger cAMP is important for various cellular processes such as proliferation, growth, migration, differentiation, and apoptosis^18–20^. These isoenzymes of cAMP-PDE expressed in several cancer cells, such as colon cancer, melanoma, prostate cancer, myeloma, pancreatic cancer, B cell lymphoma, kidney cancer, and lung cancer^18–27^. Recently, it was reported that PDE4 inhibitors possess antiproliferative effects, and inhibit the tumour cell growth of several types of cancers; thus, PDE4 inhibitors are a promising novel target for cancer therapy^18–27^. Rolipram (**B**; Figure 1)^18^^,^^22^^,^^23^, roflumilast (**C**; Figure 1)^18^^,^^22^^,^^23^, Ro-20–1724 (**D**; Figure 1)^23^, and apremilast (**E**; Figure 1)^23^ are PDE4 inhibitors that reduced the growth of colon cancer cells through regulation of the level of intracellular cAMP, leading to the induction of apoptosis. Roflumilast (**C**; Figure 1) was approved by FDA as a PDE4 inhibitor and used for the treatment of chronic obstructive pulmonary disease^26^ and was successfully tested in lung cancer and B-cell lymphoma^25^. In contrast, an increase in the level of intracellular cAMP by the inhibition of PDE4 isoenzymes leads to inhibition of the production of tumour necrosis factor-alpha (TNF-α)^28^. TNF-α is a central mediator of inflammation, and thus provides a molecular link between chronic inflammation and the development of malignancies^29–32^. In addition, TNF-α is overexpressed in various cancer cells such as liver cancer, kidney cancer, and gallbladder cancer and supports tumour growth and metastasis^29–32^. The aforementioned results indicated that the inhibition of PDE4 enzyme activity^18–27^ and the suppression of the production of TNF-α^28–32^ are an interesting target for the treatment of cancer.

Compounds containing 2-cyclopentyloxyanisole analogues are reported to be PDE4 inhibitors with anticancer activities, such as rolipram (**B**; Figure 1), roflumilast (**C**; Figure 1), and apremilast (**E**; Figure 1)^18^^,^^22^^,^^23^. Meanwhile, compounds bearing chalcone structures constitute the main building block of several natural products with potential antitumor activity, such as curcumin (**F**; Figure 1)^7^^,^^9^^,^^33^. It was reported that curcumin exerts antitumor activity against colon cancer through inhibition of the COX-2 isoenzyme^34^. Recently, curcumin was shown to have *in vitro* anti-angiogenic effects and *in vivo* anticancer activity through the inhibition of PDE isoenzymes^35^. Indeed, several compounds possessing heterocyclic core structures, such as quinazoline^2–4^, quinoline^9^^,^^10^, pyrimidine^36^, pyridine^9^, imidazole^6^, have potential antitumor activity.

Based on the aforementioned data, and to continue our efforts to develop new molecules as effective antitumor agents, we have reported (i) the synthesis of new derivatives incorporating chalcone derivatives based on the 2-cyclopentyloxyanisole core structure; (ii) the preparation of 2-cyclopentyloxyanisole bearing heterocyclic moieties such as quinazoline, quinoline, pyridine, pyrimidine, and imidazole ring systems; (iii) the synthesis of 2-cyclopentyloxyanisole bearing thioamide moieties; (iv) a comparison of the effectiveness of heterocyclic derivatives versus the chalcone and thioamide derivatives; and (v) an evaluation of the *in vitro* antitumor activity against different human cancers: liver cancer (HePG2 cell line), colon cancer (HCT-116 cell line), breast cancer (MCF-7 cell line), prostate cancer (PC3 cell line), and cervical cancer (HeLa cell line); (vi) a study of the structure–activity relationship (SAR) for the synthesised 2-cyclopentyloxyanisole structure with diverse substituent moieties regarding antitumor activities; (vii) an evaluation of the *in vitro* COX-2 and PDE4B, and TNF-α inhibitory abilities of the most promising compounds; and (viii) a molecular modelling study of the binding mode of the target molecules in the COX-2 and PDE 4 pockets.

## Experimental methods

### Chemistry

Melting points were recorded by using a Fisher-Johns melting point apparatus and were uncorrected. ^1^H NMR and ^13^C NMR spectra (500 MHz) were obtained in DMSO-d_6_ and CHCl_3_-d on a JOEL Nuclear Magnetic Resonance 500 spectrometer at Mansoura University, Faculty of Science, Egypt. Mass spectrometric analyses were performed by using a JEOL JMS-600H spectrometer at Mansoura University, Faculty of Science (Assiut, Egypt). The reaction times were determined by using a TLC technique on silica gel plates (60 F245, Merck, Kenilworth, NJ) and the spots were visualised by UV irradiation at 366 nm or 245 nm. The synthesis of 3-(cyclopentyloxy)-4-methoxybenzaldehyde (**2**) and 6-(3-(cyclopentyloxy)-4-methoxyphenyl)-4-oxo-2-thioxo-1,2,3,4-tetrahydropyrimidine-5-carbonitrile (**13**) are described elsewhere^18^^,^^37^^,^^38^.

### Synthesis of compounds 3a–c, 4a, and 4b

**Scheme 1.**
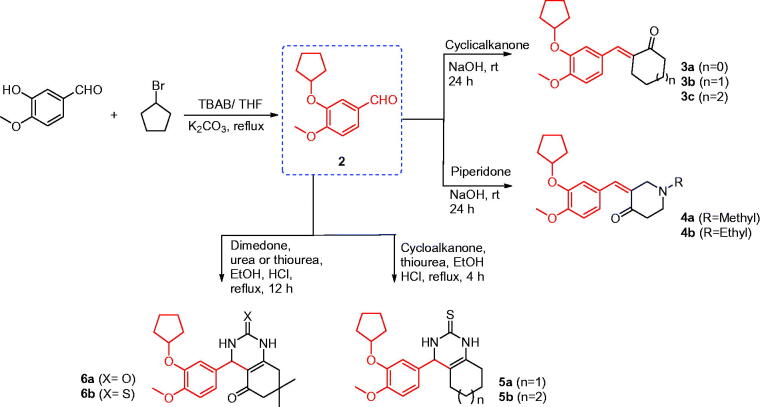
Synthesis of the designed compounds **3–6**.

To a mixture of 3-(cyclopentyloxy)-4-methoxybenzaldehyde (**2**) (1.0 mmol, 0.22 g) and cyclic ketones (3.0 mmol) in ethanol (15 ml), NaOH (2.0 mmol, 0.08 g) was added whilst stirring at 0 °C. The reaction mixture was then stirred at room temperature for 24 h, poured on crushed ice, and the obtained solid was filtered, washed with water, and recrystallised from methanol (Scheme 1).

#### 2-(3-(Cyclopentyloxy)-4-methoxybenzylidene)cyclopentanone (3a)

Yield, 65%; melting point [MP] 252–254 °C. ^1^H NMR spectrum (DMSO-d_6_), *δ*, ppm: 1.53–1.56 (2H, m), 1.62–1.65 (4H, m), 1.70–1.74 (4H, m), 1.86–1.89 (2H, m), 2.89–2.91 (2H, m), 3.87 (3H, s), 4.74–4.77 (1H, m), 7.05–7.07 (1H, d, *J* = 8.0 Hz), 7.07–7.08 (1H, d, *J* = 8.0 Hz), 7.21 (1H, s), 7.74 (1H, s). IR spectrum, *ν*, cm^−1^: 2957, 2872, 1703, 1620, 954, 642. C_18_H_22_O_3_ MS: *m/z* 287 (M^+^+1), 286 (M^+^).

#### 2-(3-(Cyclopentyloxy)-4-methoxybenzylidene)cyclohexanone (3b)

Yield, 60%; MP 245–247 °C. ^1^H NMR spectrum (DMSO-d_6_), *δ*, ppm: 1.60–1.68 (6H, m), 1.81–1.93 (8H, m), 2.91–2.93 (2H, m), 3.85 (3H, s), 4.75–4.79 (1H, m), 7.03–7.04 (1H, d, *J* = 8.1 Hz), 7.06–7.07 (1H, d, *J* = 8.0 Hz), 7.25 (1H, s), 7.77 (1H, s). IR spectrum, *ν*, cm^−1^: 2953, 2870, 1705, 1621, 951, 638. C_19_H_24_O_3_ MS: *m/z* 301 (M^+^+1), 300 (M^+^).

#### 2-(3-(Cyclopentyloxy)-4-methoxybenzylidene)cycloheptanone (3c)

Yield, 63%; MP 250–252 °C. ^1^H NMR spectrum (DMSO-d_6_), *δ*, ppm: 1.50–1.60 (3H, m), 1.80–1.81 (2H, m), 1.82–1.85 (6H, m), 1.89–1.91 (5H, m), 2.68–2.71 (2H, m), 3.86 (3H, s), 4.73–4.75 (1H, m), 6.84 (1H, s), 6.86–6.88 (1H, d, *J* = 7.9 Hz), 6.89–6.90 (1H, d, *J* = 8.0 Hz), 7.44 (1H, s). IR spectrum, *ν*, cm^−1^: 2950, 2871, 1710, 1616, 954, 639. C_20_H_26_O_3_ MS: *m/z* 315 (M^+^+1), 314 (M^+^).

#### 3-(3-(Cyclopentyloxy)-4-methoxybenzylidene)-1-methylpiperidin-4-one (4a)

Yield, 70%; MP 253–255 °C. ^1^H NMR spectrum (DMSO-d_6_), *δ*, ppm: 1.55–1.58 (2H, m), 1.64–1.73 (4H, m), 1.79–1.86 (4H, m), 2.15 (2H, s), 2.42 (3H, s), 2.91–2.95 (2H, m), 3.71 (3H, s), 4.74–4.78 (1H, q, *J* = 5.5 Hz), 6.66–6.74 (2H, m), 6.89–6.94 (2H, m). IR spectrum, *ν*, cm^−1^: 2955, 2872, 1708, 1620, 956, 640. C_19_H_25_NO_3_ MS: *m/z* 317 (M^+^+2), 316 (M^+^+1), 315 (M^+^).

#### 3-(3-(Cyclopentyloxy)-4-methoxybenzylidene)-1-ethylpiperidin-4-one (4b)

Yield, 68%; MP 249–251 °C. ^1^H NMR spectrum (DMSO-d_6_), *δ*, ppm: 1.29–1.32 (3H, t, *J* = 4.5 Hz), 1.52–1.54 (2H, m), 1.62–1.68 (4H, m), 1.81–1.85 (4H, m), 2.43–2.45 (2H, m), 2.88–2.92 (2H, m), 2.93–2.95 (2H, m), 3.73 (3H, s), 4.73–4.79 (1H, m), 6.66–6.77 (2H, m), 6.86–6.95 (2H, m). IR spectrum, *ν*, cm^−1^: 2954, 2870, 1708, 1624, 958, 644. C_20_H_27_NO_3_ MS: *m/z* 331 (M^+^+2), 330 (M^+^+1), 329 (M^+^).

### Synthesis of compounds 5a and 5b

To a solution of 3-(cyclopentyloxy)-4-methoxybenzaldehyde (**2**) (5 mmol, 1.1 g), thiourea (5 mmol, 380 mg), and cyclic ketones (7.5 mmol) in ethanol (25 ml), four drops of concentrated hydrochloric acid were added. The reaction mixture was heated under reflux for 4 h, and the solvent was evaporated under vacuum. The obtained solid was dissolved in H_2_O and the solution was neutralised with ammonia solution. The precipitated solid was filtered, washed with water, and crystallised from ethanol (Scheme 1).

#### *4-(3-(Cyclopentyloxy)-4-methoxyphenyl)-3,4,5,6,7,8-hexahydroquinazoline-2(1*H*)-thione (5a)*

Yield, 55%; MP 199–201 °C. ^1^H NMR spectrum (CHCl_3_-d), *δ*, ppm: 0.80–0.86 (4H, m), 1.20–1.25 (4H, m), 1.83–1.89 (4H, m), 1.91–1.95 (4H, m), 3.83 (3H, s), 4.67 (1H, s), 4.78–4.93 (1H, m), 6.76 (1H, s), 6.80 (1H, s), 6.82 (1H, s), 6.83–6.86 (1H, d, *J* = 8.0 Hz), 7.13–7.16 (1H, d, *J* = 8.1 Hz). IR spectrum, *ν*, cm^−1^: 3422, 3240, 2960, 2871, 1630, 1260. C_20_H_26_N_2_O_2_S MS: *m/z* 360 (M^+^+2), 359 (M^+^+1), 358 (M^+^).

#### *4-(3-(Cyclopentyloxy)-4-methoxyphenyl)-1,3,4,5,6,7,8,9-octahydro-2*H*-cyclohepta[*d*]pyrimidine-2-thione (5b)*

Yield, 52%; MP 205–207 °C. ^1^H NMR spectrum (CHCl_3_-d), *δ*, ppm: 0.83–0.88 (6H, m), 1.19–1.24 (2H, m), 1.25–1.29 (2H, m), 1.61–1.66 (4H, m), 1.82–1.93 (4H, m), 3.84 (3H, s), 4.67 (1H, s), 4.78–4.92 (1H, m), 6.76 (1H, s), 6.81 (1H, s), 6.84 (1H, s), 6.81–6.85 (1H, d, *J* = 7.9 Hz), 7.10–7.12 (1H, d, *J* = 8.0 Hz). IR spectrum, *ν*, cm^−1^: 3426, 3243, 2963, 2873, 1632, 1262. C_21_H_28_N_2_O_2_S MS: *m/z* 374 (M^+^+2), 373 (M^+^+1), 372 (M^+^).

### Synthesis of compounds 6a and 6b

To a solution of 3-(cyclopentyloxy)-4-methoxybenzaldehyde (**2**) (5 mmol, 1.1 g), urea or thiourea (5 mmol), and dimedone (7.5 mmol, 1.1 g) in ethanol (25 ml), four drops of concentrated hydrochloric acid were added. The reaction mixture was heated under reflux for 12 h and the solvent was evaporated under vacuum. The obtained solid was dissolved in H_2_O and the solution was neutralised by using ammonia solution. The precipitated solid was filtered, washed with water, and re-crystallised from DMF (Scheme 1).

#### *4-(3-(Cyclopentyloxy)-4-methoxyphenyl)-7,7-dimethyl-4,6,7,8-tetrahydroquinazoline-2,5(1*H*,3*H*)-dione (6a)*

Yield, 80%; MP 230–232 °C. ^1^H NMR spectrum (DMSO-d_6_), *δ*, ppm: 0.99 (3H, s), 1.02 (3H, s), 1.05 (1H, s), 1.54–1.58 (4H, m), 1.78–1.89 (4H, m), 2.40–2.43 (3H, t, *J* = 6.5 Hz), 3.74 (3H, s), 4.67 (1H, s), 4.72–4.76 (1H, q, *J* = 3.5 Hz), 6.67 (1H, s), 6.68 (1H, s), 6.70 (1H, s), 6.73–6.74 (1H, d, *J* = 6.5 Hz), 6.75–6.76 (1H, d, *J* = 6.5 H). IR spectrum, *ν*, cm^−1^: 3420, 3243, 2957, 2872, 1620, 1260. C_22_H_28_N_2_O_4_ MS: *m/z* 386 (M^+^+2), 385 (M^+^+1), 384 (M^+^).

#### *4-(3-(Cyclopentyloxy)-4-methoxyphenyl)-7,7-dimethyl-2-thioxo-2,3,4,6,7,8-hexahydroquinazolin-5(1*H*)-one (6b)*

Yield, 78%; MP 233–235 °C. ^1^H NMR spectrum (DMSO-d_6_), *δ*, ppm: 0.99 (3H, s), 1.01 (3H, s), 1.55 (2H, s), 1.77–1.82 (4H, m), 1.87–1.92 (4H, m), 2.44 (2H, s), 3.73 (3H, s), 4.68 (1H, s), 4.71–4.73 (1H, m), 6.66 (1H, s), 6.68 (1H, s), 6.69 (1H, s), 6.72–6.74 (1H, d, *J* = 7.5 Hz), 6.75–6.77 (1H, d, *J* = 6.5 Hz). IR spectrum, *ν*, cm^−1^: 3425, 3245, 2960, 2870, 1623, 1264. C_22_H_28_N_2_O_3_S MS: *m/z* 402 (M^+^+2), 401 (M^+^+1), 400 (M^+^).

### Synthesis of compound 7a

**Scheme 2.**
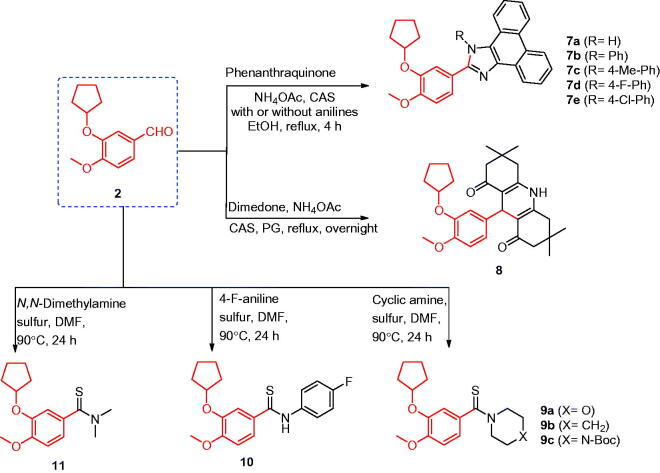
Synthesis of the designed compounds **7–11**.

A mixture of 3-(cyclopentyloxy)-4-methoxybenzaldehyde (**2**) (5 mmol, 1.1 g), 9,10-phenanthraquinone (5 mmol, 1.04 g), ammonium acetate (15 mmol, 1.17 g), and CAS or iodine (5 mol%) in ethanol (25 ml) was heated under reflux for 4 h. The reaction mixture was cooled to room temperature, poured on crushed ice, and extracted with ethyl acetate. The extract was evaporated under vacuum to yield a precipitate, which was collected and re-crystallised from acetone (Scheme 2).

#### *2-(3-(Cyclopentyloxy)-4-methoxyphenyl)-1*H*-phenanthro[9,10-*d*]imidazole (7a)*

Yield, 85%; MP 290–292 °C. ^1^H NMR spectrum (DMSO-d_6_), *δ*, ppm: 1.62 (2H, s), 1.79–1.82 (4H, m), 1.97 (2H, s), 3.84 (3H, s), 4.97 (1H, s), 7.17–7.18 (1H, d, *J* = 8.0 Hz), 7.62 (2H, s), 7.72 (2H, s), 7.87 (2H, s), 8.55–8.56 (2H, d, *J* = 6.5 Hz), 8.83–8.85 (2H, d, *J* = 7.5 Hz). ^13^C NMR spectrum (DMSO-d_6_), *δ*, ppm: 18.56, 23.68, 32.38, 55.67, 56.03, 79.95, 112.36, 112.85, 119.25, 121.88, 123.71, 125.02, 126.95, 127.07, 136.83, 147.14, 149.34, 150.92. IR spectrum, *ν*, cm^−1^: 3422, 2964, 2864, 930, 615. C_27_H_24_N_2_O_2_ MS: *m/z* 409 (M^+^+1), 408 (M^+^).

### Synthesis of compounds 7b–e

A mixture of 3-(cyclopentyloxy)-4-methoxybenzaldehyde (**2**) (5 mmol, 1.1 g), 9,10-phenanthraquinone (5 mmol, 1.04 g), ammonium acetate (15 mmol, 1.17 g), the appropriate aniline (5 mmol), and CAS or iodine (5 mol%) in ethanol (25 ml) was heated under reflux for 4 h. The formed precipitate was filtered, washed with ethanol, and crystallised from DMF (Scheme 2).

#### *2-(3-(Cyclopentyloxy)-4-methoxyphenyl)-1-phenyl-1*H*-phenanthro[9,10-*d*]imidazole (7b)*

Yield, 82%; MP 295–297 °C. ^1^H NMR spectrum (DMSO-d_6_), *δ*, ppm: 1.52 (2H, s), 1.60–1.65 (4H, m), 1.70–1.71 (2H, d, *J* = 6.5 Hz), 3.74 (3H, s), 4.49 (1H, s), 6.96–6.98 (2H, d, *J* = 8.0 Hz), 7.01–7.03 (1H, d, *J* = 8.0 Hz), 7.29–7.31 (2H, d, *J* = 7.5 Hz), 7.51–7.54 (1H, t, *J* = 7.5 Hz), 7.62–7.77 (7H, m), 8.67–8.68 (1H, d, *J* = 7.5 Hz), 8.85–8.87 (1H, d, *J* = 8.5 Hz), 8.90–8.92 (1H, d, *J* = 8.5 Hz). IR spectrum, *ν*, cm^−1^: 2960, 2869, 932, 618. C_33_H_28_N_2_O_2_ MS: *m/z* 485 (M^+^+1), 484 (M^+^).

#### *2-(3-(Cyclopentyloxy)-4-methoxyphenyl)-1-(4-methylphenyl)-1*H*-phenanthro[9,10-*d*]imidazole (7c)*

Yield, 80%; MP 291–294 °C. ^1^H NMR spectrum (DMSO-d_6_), *δ*, ppm: 1.52 (4H, s), 1.64–1.68 (4H, m), 2.07 (3H, s), 3.75 (3H, s), 4.36 (1H, s), 6.89 (1H, s), 6.98–6.70 (1H, d, *J* = 8.5 Hz), 7.12–7.14 (1H, d, *J* = 8.0 Hz), 7.32–7.37 (2H, q, *J* = 9.0 Hz), 7.50–7.54 (3H, q, *J* = 7.5 Hz), 7.56–7.58 (2H, d, *J* = 8.0 Hz), 7.65–7.68 (1H, t, *J* = 7.5 Hz), 7.74–7.77 (1H, t, *J* = 7.5 Hz), 8.66–8.67 (1H, d, *J* = 7.5 Hz), 8.85–8.86 (1H, d, *J* = 8.5 Hz), 8.90–8.92 (1H, d, *J* = 9.0 Hz). IR spectrum, *ν*, cm^−1^: 2968, 2877, 942, 632. C_34_H_30_N_2_O_2_ MS: *m/z* 499 (M^+^+1), 498 (M^+^).

#### *2-(3-(Cyclopentyloxy)-4-methoxyphenyl)-1-(4-fluorophenyl)-1*H*-phenanthro[9,10-*d*]imidazole (7d)*

Yield, 86%; MP 290–292 °C. ^1^H NMR spectrum (DMSO-d_6_), *δ*, ppm: 1.50–1.54 (2H, m), 1.60–1.64 (4H, m), 1.68–1.71 (2H, m), 3.86 (3H, s), 4.94–4.99 (1H, m), 6.85 (1H, s), 6.94–6.96 (1H, d, *J* = 7.5 Hz), 7.10–7.11 (1H, d, *J* = 7.5 Hz), 7.29–7.31 (2H, m), 7.40–7.49 (5H, m), 7.62–7.64 (1H, t, *J* = 8.0 Hz), 7.71–7.74 (1H, t, *J* = 8.0 Hz), 8.65–8.66 (1H, d, *J* = 8.5 Hz), 8.81–8.83 (1H, d, *J* = 9.0 Hz), 8.86–8.87 (1H, d, *J* = 8.5 Hz). IR spectrum, *ν*, cm^−1^: 2968, 2875, 940, 636. C_33_H_27_FN_2_O_2_ MS: *m/z* 505 (M^+^+3), 503 (M^+^+1), 502 (M^+^).

#### *2-(3-(Cyclopentyloxy)-4-methoxyphenyl)-1-(4-chlorophenyl)-1*H*-phenanthro[9,10-*d*]imidazole (7e)*

Yield, 84%; MP 294–296 °C. ^1^H NMR spectrum (DMSO-d_6_), *δ*, ppm: 1.52–1.56 (2H, m), 1.60–1.66 (4H, m), 1.69–1.73 (2H, m), 3.83 (3H, s), 4.91–4.95 (1H, m), 6.80 (1H, s), 6.94–6.96 (1H, d, *J* = 8.0 Hz), 7.12–7.14 (1H, d, *J* = 8.0 Hz), 7.25–7.29 (2H, m), 7.40–7.47 (5H, m), 7.61–7.63 (1H, t, *J* = 7.5 Hz), 7.72–7.73 (1H, t, *J* = 7.0 Hz), 8.65–8.67 (1H, d, *J* = 8.50 Hz), 8.79–8.81 (1H, d, *J* = 8.5 Hz), 8.84–8.86 (1H, d, *J* = 9.0 Hz). IR spectrum, *ν*, cm^−1^: 2965, 2873, 942, 635. C_33_H_27_ClN_2_O_2_ MS: *m/z* 520 (M^+^+2), 519 (M^+^+1), 518 (M^+^).

### Synthesis of compound 8

To a solution of 3-(cyclopentyloxy)-4-methoxybenzaldehyde (**2**) (5 mmol, 1.1 g), dimedone (10 mmol, 1.47 g), and ammonium acetate (5 mmol, 0.39 g) in propylene glycol (20 ml), CAS or iodine (5 mol%) was added. The reaction mixture was heated under reflux overnight, cooled to room temperature, and poured on crushed ice. The obtained solid was filtered, washed with water, and re-crystallised from ethanol (Scheme 2).

#### *9-(3-(Cyclopentyloxy)-4-methoxyphenyl)-3,3,6,6-tetramethyl-3,4,6,7,9,10-hexahydroacridine-1,8(2*H*,5*H*)-dione (8)*

Yield, 77%; MP 286–287 °C. ^1^H NMR spectrum (DMSO-d_6_), *δ*, ppm: 0.86 (6H, s), 0.99 (6H, s), 1.53–1.55 (2H, m), 1.63–1.67 (4H, m), 1.69–1.71 (2H, m), 1.98–2.00 (2H, d, *J* = 6.5 Hz), 2.14–2.15 (2H, d, *J* = 5.5 Hz), 2.29–2.30 (2H, d, *J* = 5.5 Hz), 2.41–2.43 (2H, d, *J* = 6.5 Hz), 3.63 (3H, s), 4.55–4.58 (1H, q, *J* = 6.0 Hz), 4.72 (1H, s), 6.60–6.62 (1H, d, *J* = 8.0 Hz), 6.69–6.71 (2H, d, *J* = 6.0 Hz), 9.26 (1H, s). ^13^C NMR spectrum (DMSO-d_6_), *δ*, ppm: 23.52, 26.39, 29.16, 31.89, 32.07, 32.28, 50.27, 55.38, 79.38, 111.44, 111.59, 115.02, 119.52, 139.68, 146.14, 147.58, 148.95, 149.07, 194.39. IR spectrum, *ν*, cm^−1^: 3420, 2968, 2872, 1735, 1738. C_29_H_37_NO_4_ MS: *m/z* 464 (M^+^+1), 463 (M^+^).

### Synthesis of compounds 9a–c, 10, and 11

A solution of 3-(cyclopentyloxy)-4-methoxybenzaldehyde (**2**) (5 mmol, 1.1 g), appropriate amine derivatives (25 mmol), and precipitated sulphur (12.5 mmol, 0.40 g) in DMF (15 ml) was heated at 90 °C for 24 h. The reaction was monitored by TLC and, after completion, was cooled to room temperature and poured on crushed ice. The formed precipitate was filtered, washed with water, and re-crystallised from methanol (Scheme 2).

#### (3-(Cyclopentyloxy)-4-methoxyphenyl)(morpholino)methanethione (9a)

Yield, 70%; MP 190–192 °C. ^1^H NMR spectrum (DMSO-d_6_), *δ*, ppm: 1.55–1.56 (2H, d, *J* = 2.5 Hz), 1.67–1.70 (4H, m), 1.86–1.87 (2H, d, *J* = 4.0 Hz), 3.58–3.59 (4H, d, *J* = 3.0 Hz), 3.75 (3H, s), 4.27 (4H, s), 4.75–4.78 (1H, t, *J* = 5.5 Hz), 6.83–6.85 (2H, t, *J* = 8.0 Hz), 6.92–6.94 (1H, d, *J* = 8.0 Hz). IR spectrum, *ν*, cm^−1^: 2956, 2848, 1516, 1223, 1163, 925, 813, 631. C_17_H_23_NO_3_S MS: *m/z* 323 (M^+^+2), 322 (M^+^+1), 321 (M^+^).

#### (3-(Cyclopentyloxy)-4-methoxyphenyl)(piperidin-1-yl)methanethione (9b)

Yield, 72%; MP 193–195 °C. ^1^H NMR spectrum (DMSO-d_6_), *δ*, ppm: 1.50–1.57 (4H, q, *J* = 6.5 Hz), 1.66–1.69 (8H, t, *J* = 6.0 Hz), 1.85–1.86 (2H, d, *J* = 4.0 Hz), 3.52–3.53 (2H, d, *J* = 5.0 Hz), 3.75 (3H, s), 4.22–4.23 (2H, d, *J* = 5.5 Hz), 4.75–4.78 (1H, t, *J* = 6.0 Hz), 6.78–6.80 (2H, d, *J* = 7.5 Hz), 6.91–6.92 (1H, d, *J* = 8.5 Hz). IR spectrum, *ν*, cm^−1^: 2955, 2846, 1512, 1225, 1166, 920, 810, 630. C_18_H_25_NO_2_S MS: *m/z* 321 (M^+^+2), 320 (M^+^+1), 319 (M^+^).

#### tert*-Butyl 4-(3-(cyclopentyloxy)-4-methoxyphenylcarbonothioyl)piperazine-1-carboxylate (9c)*

Yield, 68%; MP 191–193 °C. ^1^H NMR spectrum (DMSO-d_6_), *δ*, ppm: 1.39 (9H, s), 1.53–1.55 (2H, m), 1.68–1.70 (4H, t, *J* = 4.5 Hz), 1.86–1.87 (2H, m, *J* = 4.5 Hz), 3.30–3.33 (4H, m), 3.56–3.59 (4H, m), 3.75 (3H, s), 4.75–4.77 (1H, t, *J* = 5.5 Hz), 6.84–6.86 (2H, t, *J* = 7.0 Hz), 6.92–6.95 (1H, t, *J* = 8.0 Hz). IR spectrum, *ν*, cm^−1^: 2958, 2848, 1514, 1224, 1160, 929, 812, 633. C_22_H_32_N_2_O_4_S MS: *m/z* 421 (M^+^+1), 420 (M^+^).

#### *3-(Cyclopentyloxy)-*N*-(4-fluorophenyl)-4-methoxybenzothioamide (10)*

Yield, 75%; MP 194–196 °C. ^1^H NMR spectrum (DMSO-d_6_), *δ*, ppm: 1.55–1.58 (2H, d, *J* = 6.0 Hz) , 1.70–1.74 (4H, m), 1.91–1.94 (2H, d, *J* = 4.0 Hz), 3.81 (3H, s), 4.83–4.85 (1H, t, *J* = 5.5 Hz), 7.06–7.07 (1H, d, *J* = 7.5 Hz), 7.19–7.23 (2H, m), 7.25–7.28 (2H, m), 7.34 (1H, s), 7.49–7.50 (1H, d, *J* = 7.5 Hz), 8.48 (1H, s). IR spectrum, *ν*, cm^−1^: 2951, 2848, 1510, 1225, 1162, 921, 814, 633. C_19_H_20_FNO_2_S MS: *m/z* 347 (M^+^+2), 346 (M^+^+1), 345 (M^+^).

#### *3-(Cyclopentyloxy)-4-methoxy-*N,N*-dimethylbenzothioamide (11)*

Yield, 71%; MP 189–191 °C. ^1^H NMR spectrum (DMSO-d_6_), *δ*, ppm: 1.52–1.55 (2H, m), 1.68–1.70 (4H, t, *J* = 5.0 Hz), 1.71–1.73 (2H, m), 3.16 (3H, s), 3.46 (3H, s), 3.75 (3H, s), 4.75–4.77 (1H, t, *J* = 5.5 Hz), 6.84–6.87 (2H, m), 6.91–6.92 (1H, d, *J* = 8.5 Hz). ^13^C NMR spectrum (DMSO-d_6_), *δ*, ppm: 23.53, 32.15, 43.09, 43.98, 55.57, 79.47, 111.30, 113.04, 118.95, 135.54, 145.96, 149.78, 198.94. IR spectrum, *ν*, cm^−1^: 2956, 2851, 1514, 1229, 1159, 920, 814, 636. C_15_H_21_NO_2_S MS: *m/z* 280 (M^+^+1), 279 (M^+^).

### Synthesis of compounds 12a–c

**Scheme 3.**
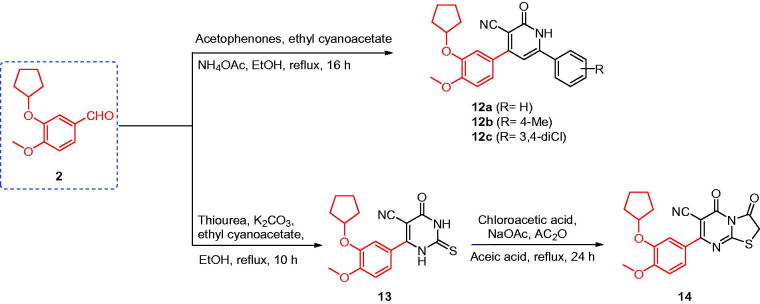
Synthesis of the designed compounds **12–14.**

A mixture of 3-(cyclopentyloxy)-4-methoxybenzaldehyde (**2**) (2 mmol, 0.44 g), the appropriate acetophenone derivatives (2 mmol), ethyl cyanoacetate (2 mmol, 0.23 g), and ammonium acetate (16 mmol, 1.24 g) in ethanol (10 ml) was heated under reflux for 16 h. The reaction mixture was cooled to room temperature, filtered, washed with ethanol, and re-crystallised from acetone (Scheme 3).

#### 4-(3-(Cyclopentyloxy)-4-methoxyphenyl)-2-oxo-6-phenyl-1,2-dihydropyridine-3-carbonitrile (12a)

Yield, 88%; MP > 300 °C; ^1^H NMR spectrum (DMSO-d_6_), *δ*, ppm: 1.57–1.58 (2H, d, *J* = 6.0 Hz), 1.71–1.76 (4H, m), 1.89–1.91 (2H, t, *J* = 11.5 Hz), 3.82 (3H, s), 4.88–4.90 (1H, m), 6.77 (1H, s), 7.10–7.12 (1H, d, *J* = 10.0 Hz), 7.30 (2H, s), 7.33 (1H, s), 7.51–7.56 (3H, m), 7.87–7.88 (2H, d, *J* = 5.0 Hz). ^13^C NMR spectrum (DMSO-d_6_), *δ*, ppm: 23.62, 32.27, 55.71, 79.71, 112.04, 114.40, 116.94, 121.41, 127.78, 128.08, 128.94, 131.13, 146.82, 151.58. IR spectrum, *ν*, cm^−1^: 3445, 2964, 2220, 1630, 1510, 1265, 810. C_24_H_22_N_2_O_3_ MS: *m/z* 387 (M^+^+1), 386 (M^+^).

#### *4-(3-(cyclopentyloxy)-4-methoxyphenyl)-2-oxo-6-(*p*-tolyl)-1,2-dihydropyridine-3-carbonitrile (12b)*

Yield, 84%; MP > 300 °C. ^1^H NMR spectrum (DMSO-d_6_), *δ*, ppm: 1.56–1.57 (2H, t, *J* = 4.0 Hz), 1.70–1.76 (4H, m), 1.88–1.92 (2H, m), 2.36 (3H, s), 3.81 (3H, s), 4.86–4.89 (1H, m), 6.74 (1H, s), 7.10–7.12 (1H, d, *J* = 8.5 Hz), 7.28–7.30 (2H, q, *J* = 4.0 Hz), 7.31 (1H, s), 7.33–7.34 (2H, d, *J* = 8.0 Hz), 7.77–7.79 (2H, d, *J* = 7.0 Hz). ^13^C NMR spectrum (DMSO-d_6_), *δ*, ppm: 20.92, 23.59, 32.24, 55.69, 79.69, 112.02, 114.39, 116.98, 121.34, 127.63, 128.14, 129.49, 141.27, 146.77, 151.52. IR spectrum, *ν*, cm^−1^: 3447, 2959, 2216, 1629, 1514, 1263, 807; C_25_H_24_N_2_O_3_ MS: *m/z* 401 (M^+^+1), 400 (M^+^).

#### 4-(3-(Cyclopentyloxy)-4-methoxyphenyl)-6-(3,4-dichlorophenyl)-2-oxo-1,2-dihydropyridine-3-carbonitrile (12c)

Yield, 81%; MP > 300 °C. ^1^H NMR spectrum (DMSO-d_6_), *δ*, ppm: 1.52–1.57 (2H, m), 1.69–1.76 (4H, m), 1.89–1.94 (2H, m), 3.82 (3H, s), 4.86–4.89 (1H, m), 7.11–7.13 (2H, d, *J* = 8.0 Hz), 7.30–7.31 (2H, d, *J* = 2.5 Hz), 7.31–7.32 (1H, d, *J* = 2.5 Hz), 7.33–7.34 (1H, d, *J* = 2.5 Hz), 7.79–7.81 (2H, d, *J* = 9.0 Hz). IR spectrum, *ν*, cm^−1^: 3443, 2964, 2222, 1635, 1508, 1268, 808. C_24_H_20_Cl_2_N_2_O_3_ MS: *m/z* 456 (M^+^+2), 454 (M^+^).

### Synthesis of compound 14

A mixture of compound **13** (1 mmol, 0.34 g), chloroacetic acid (1 mmol, 0.10 g), anhydrous sodium acetate (4 mmol, 0.33 g) in acetic anhydride (2 ml), and glacial acetic acid (10 ml) was heated under reflux for 24 h. The reaction mixture was cooled to room temperature and poured into crushed ice. The obtained solid was filtered, washed with water, and crystallised from methanol (Scheme 3).

#### *7-(3-(Cyclopentyloxy)-4-methoxyphenyl)-3,5-dioxo-2,3-dihydro-5*H*-thiazolo[3,2-*a*]pyrimidine-6-carbonitrile (14)*

Yield, 55%; MP 265–267 °C. ^1^H NMR spectrum (DMSO-d_6_), *δ*, ppm: 1.50–1.53 (2H, m), 1.69–1.72 (4H, m), 1.88–1.90 (2H, m), 3.79 (3H, s), 4.21 (2H, s), 4.76–4.79 (1H, m), 7.01 (1H, s), 7.35 (1H, s), 7.38 (1H, s). IR spectrum, *ν*, cm^−1^: 2962, 2229, 1655, 16,450, 1217, 986. C_19_H_17_N_3_O_4_S MS: *m/z* 385 (M^+^+2), 383 (M^+^).

### Biological evaluation

#### In vitro antitumor activity evaluation assay

The antitumor activity was performed by using the tetrazolium salt 3-(4,5-dimethyl-2-thiazolyl)-2,5-diphenyl-2*H*-tetrazolium bromide (MTT) assay in accordance with an established method^39^.

#### In vitro COX-2 inhibition assay

The colorimetric COX-2 inhibition assay was performed in accordance with the manufacturer’s instructions (Kit 560101, Cayman Chemical, Ann Arbour, MI)^40–42^.

#### In vitro TNF-α inhibition assay

The concentration of TNF-α was measured by human-specific sandwich enzyme-linked immunosorbent assay (ELISA) in accordance with the manufacturer’s instructions (no. 589201, Cayman Chemical, Ann Arbour, MI)^43^^,^^44^.

### Docking methodology

The molecular docking technique was performed by using MOE 2008.10, from the Chemical Computing Group Inc.^45^ in accordance with previously established methods^18^^,^^40–42^.

## Results and discussion

### Chemistry

The synthetic strategies used to obtain the target compounds are presented in Schemes 1–3. The O-alkylation of isovanillin (**1**) with bromocyclopentane was successively conducted in the presence of K_2_CO_3_ and a phase transfer catalyst tetrabutylammonium bromide (TBAB) in THF to obtain the key intermediate 3-cyclopentyloxy-4-methoxybenzaldehyde (**2**) that provided the core structure of phosphodiesterase-4 inhibitors^37^. Tetrabutylammonium bromide successively exhibited the character of phase transfer catalyst in an environmentally friendly procedure under mild conditions^37^.

### Synthesis of compounds 3–6

First, the cyclocondensation of 3-cyclopentyloxy-4-methoxybenzaldehyde (**2**)^37^ with cyclic ketones in the ethanolic solution of sodium hydroxide afforded chalcones **3a**–**c** and **4a**,**b** in good yields (Scheme 1). In addition, the one-pot cyclocondensation reaction of **2** with the cyclic ketone (cyclohexanone/cycloheptanone/dimedone) and urea or thiourea in ethanol containing few drops of concentrated hydrochloric acid yielded the quinazoline derivatives **5a,b** and **6a,b**^46^, as shown in Scheme 1.

### Synthesis of compounds 7–11

The synthesis of imidazole via multicomponent reactions (MCRs) was achieved through the cyclocondensation of 1,2-diketone, an aldehyde, and ammonium acetate using a catalytic amount of ceric ammonium sulphate (CAS) or molecular iodine^47^^,^^48^ (Scheme 2). Thus, a one-pot synthesis achieved phenanthroimidazole derivatives **7a–e** in good yield via the cyclocondensation of 9,10-phenanthraquinone, 3-cyclopentyloxy-4-methoxybenzaldehyde (**2**), and ammonium acetate in the presence of 5% mole of iodine or CAS. Furthermore, acridinedione **8** was prepared by a one-pot, three-component cyclocondensation reaction of 3-cyclopentyloxy-4-methoxybenzaldehyde (**2**), 1,3-dicarbonyl compound (dimedone), and ammonium acetate in the presence of a catalytic amount of 5% CAS using polyethylene glycol (PEG) as a solvent^49^. Thioamides **9a**–**c**, **10**, and **11** were synthesised^50^ by the reaction of elemental sulphur (S_8_), 3-cyclopentyloxy-4-methoxybenzaldehyde (**2**), and secondary amines, such as piperidine, morpholine, N-Boc-piperazine, and dimethylamine, or primary amines, such as 4-fluoroaniline in dimethylformamide (DMF), under heating condition.

### Synthesis of compounds 12–14

The MCRs of 3-cyclopentyloxy-4-methoxybenzaldehyde (**2**), ethyl cyanoacetate, an appropriate acetophenone, and ammonium acetate in EtOH at reflux temperature gave pyridine-3-carbonitrile derivatives **12a**–**c** in good yield. In contrast, the reaction of 3-cyclopentyloxy-4-methoxybenzaldehyde (**2**) with ethyl cyanoacetate and thiourea in an ethanolic solution of K_2_CO_3_ afforded 6-(3-(cyclopentyloxy)-4-methoxyphenyl)-4-oxo-2-thioxo-1,2,3,4-tetrahydropyrimidine-5-carbonitrile (**13**)^18^^,^^38^. Compound **13** was cyclised with chloroacetic acid in the presence of acetic anhydride and anhydrous sodium acetate in glacial acetic acid to yield thiazolo[3,2-*a*]pyrimidine-3,5-dione derivative **14**^51^ (Scheme 3).

### Biological evaluation

#### Antitumor evaluation using the MTT assay

Compounds **3a**–**c**, **4a**,**b**, **5a**,**b**, **6a**,**b**, **7a**–**e**, **8**, **9a**–**c**, **10**, **11**, **12a**–**c**, **13**, and **14** were screened for their *in vitro* antitumor activity by using the standard 3-(4,5-dimethylthiazol-2-yl)-2,5-diphenyltetrazolium bromide (MTT) assay against five human cancers: HePG2, HCT-116, MCF-7, PC3, and HeLa cell lines^39^. The antitumor activities of the synthesised compounds **3**–**14** and the reference drugs, celecoxib, afatinib, and doxorubicin, are shown in Table 1^8–10^. Compounds **3a**–**c**, incorporating the cycloalkanone core, possessed strong to weak antitumor activity against some of the investigated cell lines (IC_50_ ≅ 19.34–95.96 μM). Interestingly, the replacement of the cycloalkanone moieties, such as in compounds **3a–c**, with a piperidin-4-one fragment, such as compound **4a**,**b**, resulted in a sharp increase in antitumor activity (IC_50_ ≅ 4.38–14.32 μM) against all of the investigated five cell lines, compared with the reference drug, celecoxib (IC_50_ ≅ 25.6–36.08 μM), afatinib (IC_50_ values of 5.4–11.4 μM), and doxorubicin (IC_50_ ≅ 4.17–8.87 μM).

**Table 1.** *In vitro* antitumor activity of the designed compounds, celecoxib, afatinib, and doxorubicin against human tumour cells.

Compound no.	IC_50_ (µM)^a^				
	HePG2	HCT-116	MCF-7	PC3	HeLa
**3a**	95.96 ± 5.2	>100	56.14 ± 2.6	51.43 ± 3.0	59.12 ± 3.8
**3b**	53.87 ± 3.7	80.56 ± 3.9	23.81 ± 1.5	19.34 ± 1.8	26.11 ± 1.9
**3c**	86.90 ± 4.5	93.46 ± 5.1	>100	>100	81.65 ± 4.7
**4a**	6.04 ± 0.5	4.38 ± 0.4	5.13 ± 0.3	9.18 ± 0.8	7.24 ± 0.7
**4b**	10.96 ± 1.1	9.48 ± 0.8	7.18 ± 0.8	14.32 ± 1.2	8.56 ± 0.9
**5a**	73.41 ± 3.7	66.48 ± 3.8	92.37 ± 5.2	78.95 ± 4.1	84.26 ± 4.6
**5b**	59.08 ± 3.5	61.13 ± 3.6	81.20 ± 4.3	55.17 ± 3.1	46.29 ± 3.0
**6a**	18.53 ± 1.7	30.49 ± 1.8	28.62 ± 1.6	27.44 ± 2.1	19.12 ± 1.7
**6b**	16.05 ± 1.4	25.41 ± 1.7	10.27 ± 1.1	17.95 ± 1.6	13.49 ± 1.4
**7a**	78.21 ± 4.4	90.34 ± 4.9	89.79 ± 4.3	>100	77.64 ± 4.6
**7b**	13.68 ± 1.2	19.67 ± 1.4	11.85 ± 1.3	22.89 ± 1.9	17.18 ± 1.5
**7c**	57.08 ± 3.9	81.19 ± 4.2	65.32 ± 3.4	68.06 ± 3.5	53.18 ± 3.7
**7e**	29.89 ± 2.1	44.82 ± 2.3	42.41 ± 2.2	46.97 ± 2.7	38.05 ± 2.5
**8**	41.82 ± 3.0	70.52 ± 3.5	60.48 ± 2.8	55.82 ± 3.2	43.47 ± 2.9
**9a**	32.87 ± 2.3	48.13 ± 2.4	35.17 ± 1.9	29.23 ± 2.3	37.50 ± 2.5
**9b**	24.85 ± 1.9	39.07 ± 2.2	37.09 ± 2.0	31.50 ± 2.4	28.37 ± 2.3
**19c**	36.27 ± 2.5	52.87 ± 2.7	48.93 ± 2.3	33.39 ± 2.6	40.61 ± 2.8
**10**	49.86 ± 3.5	79.12 ± 3.8	64.10 ± 3.1	47.32 ± 2.9	52.50 ± 3.7
**11**	91.23 ± 4.8	96.79 ± 5.5	94.27 ± 4.7	88.63 ± 5.0	90.89 ± 4.9
**12a**	45.24 ± 3.4	76.05 ± 3.6	71.63 ± 3.9	79.83 ± 4.0	65.72 ± 4.1
**12b**	38.14 ± 2.8	67.74 ± 3.5	58.28 ± 2.7	61.45 ± 3.3	45.69 ± 3.2
**12c**	59.63 ± 4.0	83.42 ± 4.3	66.07 ± 3.7	73.48 ± 3.8	62.76 ± 3.9
**13**	8.71 ± 0.7	7.66 ± 0.6	6.93 ± 0.5	11.45 ± 1.1	5.86 ± 0.6
**14**	20.11 ± 1.8	34.93 ± 1.9	9.62 ± 0.9	15.31 ± 1.3	12.48 ± 1.2
**Celecoxib**	25.6 ± 2.3	29.54 ± 2.1	31.28 ± 2.5	30.69 ± 2.7	36.08 ± 2.8
**Afatinib**	5.4 ± 0.25	11.4 ± 1.26	7.1 ± 0.49	7.7 ± 0.57	6.2 ± 0.67
**DOX**	4.50 ± 0.2	5.23 ± 0.3	4.17 ± 0.2	8.87 ± 0.6	5.57 ± 0.4

DOX: doxorubicin.

^a^ IC_50_, compound concentration required to inhibit tumour cell proliferation by 50% (mean ± SD, *n* = 3). IC_50_, (μM): 1–10 (very strong), 11–25 (strong), 26–50 (moderate), 51–100 (weak), and above 100 (non-cytotoxic). Compound **7d** had an IC_50_ of >100 µM.

Moreover, the introduction of quinazoline-2-thione or pyrimidine-2-thione moieties, instead of a piperidin-4-one moiety, as in compounds **5a**,**b**, resulted in a sharp decrease in antitumor activity against all the investigated five cancer cell lines, with IC_50_ values in the range 46.29–92.37 μM. In contrast, the replacement of the quinazoline-2-thione fragment, as in compound **5a**, with quinazoline-2,5-dione and 2-thioxo-quinazolin-5-one fragments at the same position, such as compounds **6a** and **6b**, resulted in a sharp increase in antitumor activity against all the investigated cancer cell lines, for HePG2 (IC_50_ values of 18.53 and 16.05 μM, respectively), HCT-116 (IC_50_ values of 30.49 and 25.41 μM, respectively), MCF-7 (IC_50_ values of 28.62 and 10.27 μM, respectively), PC3 (IC_50_ values of 27.44 and 17.95 μM, respectively), and HeLa (IC_50_ values of 19.12 and 13.49 μM, respectively), compared with celecoxib (IC_50_ values of 25.6, 29.54, 31.28, 30.69, and 36.08 μM, respectively), afatinib (IC_50_ values of 5.4, 11.4, 7.1, 7.7, and 6.2 μM, respectively), and doxorubicin (IC_50_ values of 4.50, 5.23, 4.17, 8.87, and 5.57 μM, respectively).

Moreover, weak antitumor activity against some of the tested cancer cell lines was exhibited by some polycyclic derivatives incorporating imidazole and quinoline ring systems, such as compounds **7a** and **7c** (IC_50_ ≅ 53.18–90.34 μM), whereas compounds **7e** and **8** showed moderate antitumor activity against some selected cancer cell lines (IC_50_ ≅ 29.8–46.97 μM). Unexpectedly, derivative **7b** showed a sharp increase in antitumor activity compared with the structural analogues **7a**, **c**, **d**, and **8**, with IC_50_ values of 13.68, 19.67, 11.85, 22.89, and 17.18 μM against HeG2, HCT-116, MCF-7, PC3, and HeLa cancer cell lines, respectively.

In contrast, the introduction of thioamide fragments in the 2-cyclopentyloxyanisole scaffold resulted in variable antitumor activity against the tested cancer cell lines; for example, compounds **9a**–**c** showed strong to moderate antitumor activity (IC_50_ ≅ 24.85–48.93 μM) in comparison with thioamide **10** (IC_50_ ≅ 47.32–79.12 μM) and **11** (IC_50_ ≅ 88.63–96.79 μM). Furthermore, replacement of the thioamide moiety with a pyridine fragment, such as in compounds **12a**–**c**, retained the antitumor activity against all cancer cell lines, as indicated by their IC_50_ values in the range 38.14–83.42 μM. In contrast, the 2-cyclopentyloxyanisole scaffold bearing the pyrimidine ring system, such as compounds **13** and **14**, exhibited strong antitumor activities against the cancer cell lines tested (IC_50_ ≅ 5.86–20.11 μM). In brief, the compounds **4a**, **4b**, **7b**, and **13** exhibited the strongest antitumor activities among the designed compounds against the HeG2, HCT-116, MCF-7, PC3, and HeLa cancer cell lines (IC_50_ ≅ 4.38–22.89 μM).

#### Structure–activity relationship of antitumor activity

According to the aforementioned antitumor activity, the SARs for the designed compounds indicated the following. (i) *N*-Methylpiperidin-4-one derivative **4a** and *N*-ethylpiperidin-4-one derivative **4b** exhibited higher antitumor activity (IC_50_ ≅ 4.38–14.32 μM) than the corresponding cycloalkanones **3a**–**c** (IC_50_ ≅ 19.34 to >100 μM). It was clear that the derivative with *N*-methylpiperidin-4-one **4a** had greater antitumor activity against all tested cancer cell lines (IC_50_ ≅ 4.38–9.18 μM) than the *N*-ethylpiperidin-4-one derivative **4b** (IC_50_ ≅ 7.18–14.32 μM). (ii) Similarly, cyclohexanone derivative **3b** exhibited greater antitumor activity against MCF-7 (IC_50_=23.81 μM), PC3 (IC_50_=19.34 μM), and HeLa (IC_50_=26.11 μM) cancer cells than cyclopentanone derivative **3a** (IC_50_ ≅ 51.43 to >100 μM), and cycloheptanone derivative **3c** (IC_50_ ≅ 81.65 to >100 μM). (iii) Compounds incorporating a quinazoline fragment, such as quinazoline-2,5(1H,3H)-dione derivative **6a** (IC_50_ ≅ 18.53–30.49 μM) and 2-thioxoquinazolin-5(1H)-one derivative **6b** (IC_50_ ≅ 10.27–25.41 μM) showed higher antitumor activity than the corresponding derivatives quinazoline-2(1H)-thione **5a**, and pyrimidine-2-thione **5b** (IC_50_ ≅ 46.29–92.37 μM). (iv) The 2-cyclopentyloxyanisole scaffold bearing the bulky polycyclic 1H-phenanthro[9,10-d]imidazoles **7a,c,d,e** (IC_50_ ≅ 29.89 to >100 μM), and acridine-1,8(2H,5H)-dione **8** (IC_50_ ≅ 41.82–70.52 μM) showed lower antitumor activity than the corresponding 2-cyclopentyloxyanisole scaffold bearing quinazoline moiety **6a,b** (IC_50_ ≅ 10.27–30.49 μM). Interestingly, the derivative **7b** with the phenyl ring at position 1 of 1H-phenanthro[9,10-d]imidazole core structure (IC_50_ ≅ 11.85–22.89 μM) showed a sharp increase in antitumor activity in comparison with derivatives **7a,c,d,e** and had approximately similar activity with compound **6b** (IC_50_ ≅ 10.27–25.41 μM). (v) The antitumor activities of the 2-cyclopentyloxyanisole scaffold bearing a methanethione fragment, such as *N*-(4-fluorophenyl)benzothioamide derivative **10** (IC_50_ ≅ 47.32–79.12 μM) and *N*,*N-*dimethylbenzothioamide derivative **11** (IC_50_ ≅ 88.63–96.79 μM), were less potent than derivatives that contained morpholinomethanethione derivative **9a** (IC_50_ ≅ 29.23–48.13 μM), piperidin-1-ylmethanethione derivative **9b** (IC_50_ ≅ 24.85–39.07 μM), and *tert*-butyl piperazine-1-carboxylate derivative **9c** (IC_50_ ≅ 33.39–52.87 μM). (vi) The pyrimidine derivatives, 4-oxo-2-thioxo-1,2,3,4-tetrahydropyrimidine-5-carbonitrile derivative **13** and 3,5-dioxo-2,3-dihydro-5H-thiazolo[3,2-a]pyrimidine-6-carbonitrile derivative **14**, had potent antitumor activities (IC_50_ ≅ 5.86–20.11 μM) compared with that of the pyridine derivatives, 6-aryl-2-oxo-1,2-dihydropyridine-3-carbonitriles **12a–c**, which have moderate to weak antitumor activity (IC_50_ ≅ 38.14–83.42 μM) against all tested cancer cells. Briefly, the structure–activity correlation of antitumor activity revealed that compounds **4a**, **4b**, **6b**, **7b**, **13**, and **14** were the most active compounds, whereas compound **7d** was the only derivative that had no antitumor activity against any of the tested cancer cell lines.

#### COX-2 inhibition assay

Several compounds that possess COX-2 inhibition activity have shown potent antitumor activities that may be attributable to the role of the COX-2 enzyme in cell proliferation^8^^,^^14–17^. Accordingly, the four compounds (**4a**, **4b**, **7b**, and **13**) that exhibited the greatest antitumor activity, as well as celecoxib (used as the reference drug) were subjected to colorimetric COX-2 inhibition assays by using a COX-2 assay kit (catalogue no. 560101, Cayman Chemicals Inc., Ann Arbour, MI). The measured IC_50_ (μM) values are shown in Table 2, and are expressed as the means of three acquired determinations^40–42^. The IC_50_ values of celecoxib for COX-2 inhibition are found to be 0.68 μM. It is clear that compounds **4b** and **13** were found to be the most active inhibitors of COX-2, with IC_50_ values of 1.08 and 1.88 μM, respectively, whereas compound **4a** exhibited lower COX-2 inhibitory effect with an IC_50_ value of 3.34 μM. In contrast, compound **7b** showed a very low inhibitory effect, with an IC_50_ value for COX-2 inhibition of 24.02 μM. Briefly, a small heterocyclic substituent on the 2-cyclopentyloxyanisole core, such as the piperidine ring in compounds **4a** and **4b** and the pyrimidine ring in compound **13,** exhibited higher COX-2 inhibition in comparison with the polycyclic 1H-phenanthro[9,10-d]imidazole in compound **7b**. The reduced inhibitory effect of compound **7b** on COX-2 may be attributed to the bulkiness of the polycyclic system, which interferes with the COX-2 binding interactions.

**Table 2.** *In vitro* inhibitory effects of COX-2, PDE-4B, and TNF-α of the antitumor compounds **4a, 4b**, **7b**, and **13**.^a^

Compound no.	IC_50_ (µM)^a^		
	COX-2 inhibition	PDE-4B inhibition	TNFα inhibition
**4a**	3.34	5.62	2.012
**4b**	1.08	11.62	17.67
**7b**	24.02	5.65	13.94
**13**	1.88	3.98	6.72
**Roflumilast**	–	1.55	–
**Celecoxib**	0.68	–	6.44

^a^ IC_50_ value is the compound concentration required to produce 50% inhibition.

#### PDE-4B enzyme assay

Compounds that inhibit PDE4 were recently shown to possess effective antitumor activities owing to the overexpression of PDE4 in cancer and its role in cell proliferation and tumour cell growth^18–27^. The compounds that were the most active antitumor agents, such as compounds **4a**, **4b**, **7b**, and **13**, were subjected to a PDE4B inhibition assay using roflumilast as a reference drug; the IC_50_ values are presented in Table 2. Compound **13** showed the highest inhibition against PDE4B, with an IC_50_ value of 3.98 μM comparable to that of the reference drug roflumilast (IC_50_=1.55 μM), whereas compounds **4a** and **7b** were found have moderate activity, with IC_50_ values of 5.62 and 5.65 μM, respectively. Compound **4b** possessed the lowest activity against PDE4B, with an IC_50_ value of 11.62 μM. From the structural study of the tested derivatives, including **4a**, **4b**, **7b**, and **13**, we concluded that the 2-cyclopentyloxyanisole scaffold bearing a cyanopyrimidine fragment, such as compound **13**, increased the PDE4B inhibitory activity in comparison with other heterocyclic derivatives.

#### TNF-α inhibition assay

TNF-α has been reported as a target for cancer treatment; presently, TNF antagonists are under clinical investigation in phase I and II trials as single agents for cancer therapy^29–32^. Accordingly, compounds **4a**, **4b**, **7b**, and **13**, which are the most active antitumor agents, were subjected to the TNF-α inhibition assay using celecoxib as a reference drug^43^; the IC_50_ values are presented in Table 2. Compound **4a** possessed potent TNF-α inhibitory effect, with an IC_50_ value of 2.01 µM, comparable with the reference drug celecoxib (IC_50_=6.44 µM), whereas compound **13** was found to be an effective inhibitor, with an IC_50_ value of 6.72 µM, similar to the TNF-α inhibitory effect of the reference drug celecoxib (IC_50_=6.44 µM). In contrast, compounds **4b** and **7b** were the least active derivatives, with IC_50_ values of 17.67 and 13.94 µM, respectively.

### Molecular modelling analysis

Molecular modelling and docking analysis is an important technique used to establish the theoretical interaction between the bioactive molecules and the target enzyme and receptor to understand their binding mode^52^^,^^53^. Therefore, a molecular docking analysis was performed by using MOE 2008.10 software and viewer utility (Chemical Computing Group Inc., Montreal, Canada) in accordance with the standard MOE procedure^45^.

### Docking with the COX-2 isoenzyme

**Figure 2.**
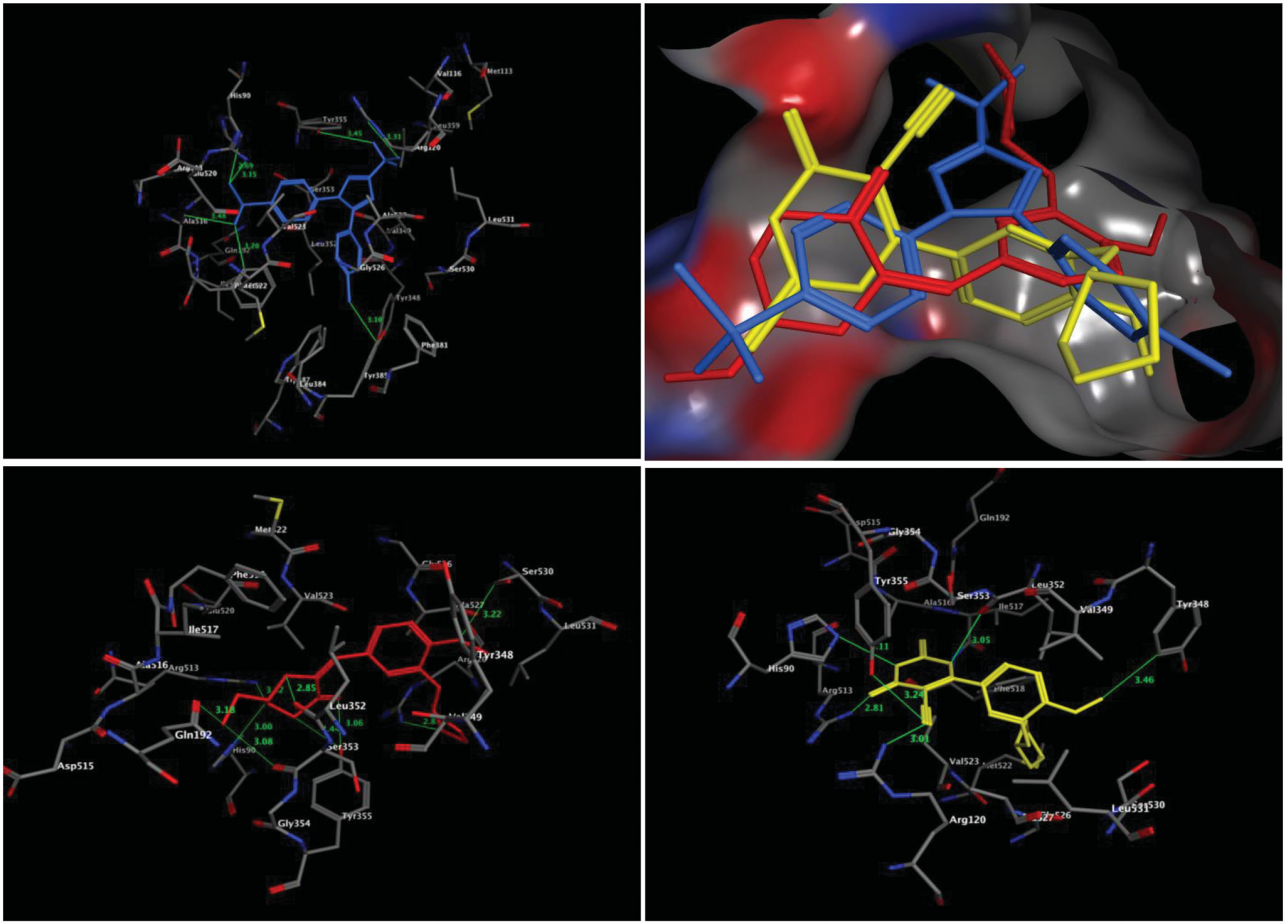
Three-dimensional (3D) orientation of the docked ligand SC-558 (upper left panel); docked compounds **4b** (lower left panel), and **13** (lower right panel) in the active pocket of the COX-2 enzyme (H bond interactions are shown as green lines). Upper right panel showed the alignment of SC-558, **4b**, and **13** in the active pocket of the COX-2 enzyme.

The molecular interaction of the most active compounds, **4b** and **13**, with the COX-2 isoenzyme was studied by molecular docking. The crystal structure of the COX-2 isoenzyme interacting with its inhibitor SC-558 was obtained from the RSC Protein Data Bank (PDB code: 1CX2)^54^. The putative binding site of the COX-2 isoenzyme (Figure 2), which is responsible for the hydrogen bonds and hydrophobic interactions with its inhibitors, consists of key amino acid residues, such as Arg510, Gln192, Arg120, Tyr355, His90, Val523, Ser353, and Ile517. The docking procedure was validated by including the bound inhibitor SC-558 for a one-ligand run docking calculation.

The bound ligand SC-558 exhibited two types of hydrogen bonds, classical and non-classical hydrogen bonds. Four classical hydrogen bonding interactions were observed with Arg513, His90, Arg120, and Tyr355. In addition, three non-classical hydrogen bonds connected the amino acids Tyr385, Phe518, and Ala516, and the benzenesulfonamide and 4-bromophenyl fragments of SC-558 through CH–O and CH–Br interactions (Figure 2, upper panel).

Interestingly, compounds **4b** and **13**, which were the most active COX-2 inhibitors, were placed in the same binding site of the inhibitor SC-558 (Figure 2). Compound **4b**, which has nearly similar COX-2 inhibition activity as celecoxib, accommodated an orientation within the COX-2 binding site (Figure 2, left lower panel), in which the *N*-ethylpiperdine-4-one fragment was located towards the secondary pocket of the COX-2 isoenzyme and interacted with the amino acid residues of Arg513, His90, Leu352, and Gln192. In general, when compound **4b** was docked into the enzyme pocket, nine hydrogen bonds were formed with the surrounding amino acids lining the pocket. One of these interactions was a classical hydrogen bond between the carbonyl (C=O) group of the N-ethylpiperdine-4-one fragment and the OH group of the Tyr355 residue (3.06 Å). Moreover, eight non-bonding interactions, namely non-classical hydrogen bonds were formed, among the two bonds of the OH of the Tyr355 residue, and the C=O of the Leu352 residue with the CH_2_ of the piperdine-4-one moiety (3.44 Å, and 2.85 Å, respectively), and among two more bonds among the C=O fragments of the Gln192 and Ser353 residues and the CH_3_ moiety of *N*-ethylpiperdine-4-one (3.18 Å and 3.08 Å, respectively). The amino acid residues Arg513 and His90 formed additional two bonds between their HN groups and the CH_2_ of the piperdine-4-one ring (3.52 Å and 3.00 Å, respectively). Finally, the amino acid residues Arg120 and Ser530 formed two non-classical hydrogen bonds with the cyclopentyl and methoxyl moieties of the anisole core structure (NH–CH_2_, 2.87 Å; and CH_2_–OCH_3_, 3.22 Å, respectively). The overall outcome of the molecular docking of compound **4b**, with respect to non-classical hydrogen bonds, showed that compound **4b** had more hydrophobic interactions with the protein than the bound ligand SC-558.

The molecular docking analysis of compound **13** showed that the 4-oxo-2-thioxo-1,2,3,4-tetrahydropyrimidine-5-carbonitrile moiety was the main fragment responsible for COX-2 activity, which interacted with the surrounding amino acid residues of the active pocket of the COX-2 isoenzyme, such as Arg513, His90, Tyr348, Tyr355, and Arg120 (Figure 2, right lower panel). Four classical and one non-classical hydrogen bonding interactions were formed between the abovementioned amino acid residues and compound **13**. The nitrile group (CN) of compound **13** formed two classical hydrogen bonds with Arg120 (3.01 Å) and Tyr355 (3.24 Å), whereas the 4-oxo-tetrahydropyrimidine ring system interacted with amino acid residues Arg513 and His90 through two classical hydrogen bonds (2.81 Å and 3.11 Å, respectively). The final interaction was the hydrophobic interaction between Tyr348 and the methoxyl moiety of anisole through a CH_2_–*π* bond, with a non-bonding distance of 3.46 Å.

### Docking with the PDE4B enzyme

**Figure 3.**
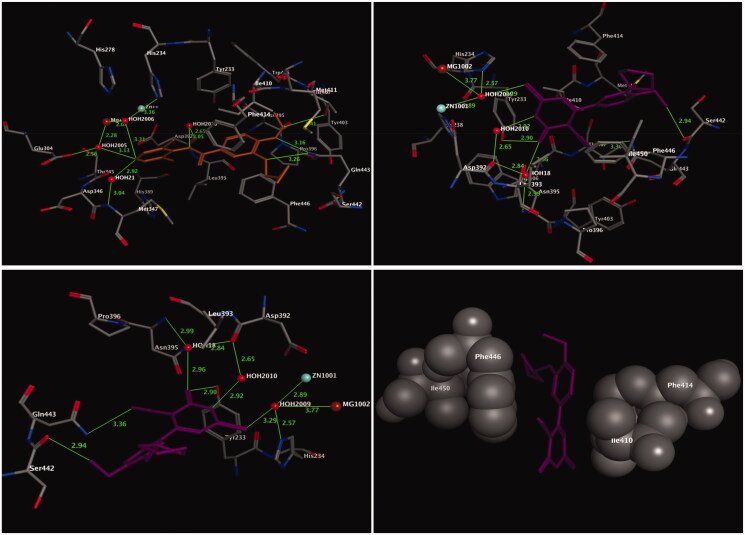
Three-dimensional (3D) orientation of the docked roflumilast (upper left panel); docked compound **13** (upper right panel), in the active pocket of the PDE4B enzyme (H bond interactions are shown as green lines). Lower left panel showed near picture of compound **13** in the active pocket of the PDE4B enzyme. Lower right panel showed the hydrophobic interactions of compound **13** in the active pocket of the PDE4B enzyme.

The binding mode of the most active compound, **13**, within the PDE4B enzyme was analysed by using molecular docking. The crystal structure of the PDE4B enzyme bound with its inhibitor roflumilast was obtained from the RSC Protein Data Bank (PDB code: 1XMU)^55^. The binding site of the PDE4B enzyme (Figure 3), which is responsible for the formation of coordination bonds, hydrogen bonds, and hydrophobic interactions with its inhibitor roflumilast, has three main sites for interaction: the solvent-filled metal coordination pocket, including both zinc and magnesium; the conserved residue Gln443; and the hydrophobic pocket. The amino acid residues Phe414, Ile410, Phe446, and Ile450 were the key residues that formed the tunnel, and were responsible for the accommodation of the hydrophobic interaction with the bound inhibitor, roflumilast. The molecular docking procedure was validated by performing a one-ligand run docking calculation for the bound inhibitor roflumilast. The results of the docking calculation of compound **13** are presented in Figure 3 (upper right panel). From the docking results, it was clear that the 2-cyclopentyloxyanisole scaffold and the pyrimidine ring adapted for hydrophobic recognition at the binding cavity lining with the amino acid residues Phe414, Ile410, Phe446, and Ile450 (Figure 3, lower right panel), similar to the bound inhibitor roflumilast (Figure 3, upper left panel). In contrast, the methoxyl group of the 2-cyclopentyloxyanisole scaffold formed a non-classical hydrogen bond with Ser442 (2.94 Å), whereas the conserved residue Gln443 interacted with the pyrimidine ring system through the nitrile moiety by the formation of hydrogen bond with a distance of 3.36 Å (Figure 3, lower left panel). Moreover, the pyrimidine ring projected towards the metal-coordinating site filled with water molecules. Accordingly, the thione (C=S) moiety of the pyrimidine ring is coordinated with Zn and Mg ions, mediated by HOH2009, and formed a hydrogen bond with the amino acid residue His234. Meanwhile, the carbonyl oxygen (C=O) of the pyrimidine formed one hydrogen bond with Tyr233 (2.90 Å) and another two hydrogen bonds with the amino acid residues Asn395 and Asp392, mediated by HOH18. Finally, the internal NH group of pyrimidine ring was adapted to form a hydrogen bond with Asp392 mediated by HOH18.

Briefly, in comparison of compound **13** with the bound inhibitor roflumilast, both compounds accommodated approximately similar interactions at the hydrophobic clamp site (Phe414, Ile410, Phe446, and Ile450) and the metal coordination site.

## Conclusions

A series of compounds incorporating 2-cyclopentyloxyanisole scaffold bearing a variety of ring systems—cycloalkanones **3a–c** and **4a–b**, quinazolines **5a–b** and **6a–b**, fused imidazoles **7a–e**, fused quinoline **8**, thioamides **9a–c, 10**, and **11**, pyridines **12a–c**, and pyrimidines **13** and **14** was synthesised. These compounds were evaluated for their *in vitro* antitumor activity in five human cancer cell lines: HePG2, HCT-116, MCF-7, PC3, and HeLa. The antitumor activity of compounds **4a**, **4b**, **6b**, **7b**, **13**, and **14** indicated that these derivatives were the most potent antitumor agents among the tested compounds, with IC_50_ values of 5.13–17.95 μM in the tested cancer cell lines. The antitumor results of the synthesised compounds were comparable with the reference drug celecoxib (IC_50_ values of 25.6–36.08 μM), afatinib (IC_50_ values of 5.4–11.4 μM), and doxorubicin (IC_50_ values of 4.17–8.87 μM). In addition, the compounds that were most active as antitumor agents, **4a**, **4b**, **7b**, and **13**, were assayed for their ability to inhibit COX-2, PDE4B, and TNF-α. The results indicated that compounds **4b** and **13** exhibited effective COX-2 inhibitory activity, with IC_50_ values of 1.08 and 1.88 μM, respectively, which were comparable with celecoxib (IC_50_=6.44 μM). In addition, compounds **4a** and **13** inhibited the PDE4B enzyme, with an IC_50_ value of 5.62 and 3.98 μM, respectively, which was comparable with roflumilast (IC_50_=1.55 μM), whereas these compounds had potent TNF-α inhibitory effect, with IC_50_ values of 2.01 and 6.72 μM, respectively, which were comparable with the reference drug celecoxib (IC_50_=6.44 μM). Compounds **4b** and **13** were docked into the COX-2 and PDE4B binding sites and exhibited similar binding characteristics to that of bound inhibitor SC-558 for the COX-2 enzyme and the bound inhibitor roflumilast for the PDE4B enzyme.
